# Therapeutic Sequencing in ALK^+^ NSCLC

**DOI:** 10.3390/ph14020080

**Published:** 2021-01-21

**Authors:** Mei Elsayed, Petros Christopoulos

**Affiliations:** 1 Department of Thoracic Oncology, Thoraxklinik and National Center for Tumor Diseases (NCT) at Heidelberg University Hospital, 69126 Heidelberg, Germany; mei.elsayed@med.uni-heidelberg.de; 2 Translational Lung Research Center Heidelberg (TLRC-H), Member of the German Center for Lung Research (DZL), 69126 Heidelberg, Germany

**Keywords:** ALK^+^ non-small-cell lung cancer, tyrosine kinase inhibitors, *EML4-ALK* fusion variant 3, chemotherapy, sequential therapies

## Abstract

Anaplastic lymphoma kinase-rearranged non-small-cell lung cancer (ALK^+^ NSCLC) is a model disease for the use of targeted pharmaceuticals in thoracic oncology. Due to higher systemic and intracranial efficacy, the second-generation ALK tyrosine kinase inhibitors (TKI) alectinib and brigatinib have irrevocably displaced crizotinib as standard first-line treatment, based on the results of the ALEX and ALTA-1L trials. Besides, lorlatinib and brigatinib are the preferred second-line therapies for progression under second-generation TKI and crizotinib, respectively, based on the results of several phase II studies. Tissue or liquid rebiopsies at the time of disease progression, even though not mandated by the approval status of any ALK inhibitor, are gaining importance for individualization and optimization of patient management. Of particular interest are cases with off-target resistance, for example *MET*, *HER2* or *KRAS* alterations, which require special therapeutic maneuvers, e.g., inclusion in early clinical trials or off-label administration of respectively targeted drugs. On the other hand, up to approximately half of the patients failing TKI, develop anatomically restricted progression, which can be initially tackled with local ablative measures without switch of systemic therapy. Among the overall biologically favorable ALK^+^ tumors, with a mean tumor mutational burden uniquely below 3 mutations per Mb and the longest survival among NSCLC currently, presence of the *EML4-ALK* fusion variant 3 and/or *TP53* mutations identify high-risk cases with earlier treatment failure and a need for more aggressive surveillance and treatment strategies. The potential clinical utility of longitudinal ctDNA assays for earlier detection of disease progression and improved guidance of therapy in these patients is a currently a matter of intense investigation. Major pharmaceutical challenges for the field are the development of more potent, fourth-generation TKI and effective immuno-oncological interventions, especially ALK-directed cell therapies, which will be essential for further improving survival and achieving cure of ALK^+^ tumors.

## 1. The New Era of Second-Generation ALK Inhibitors

**Figure 1:**
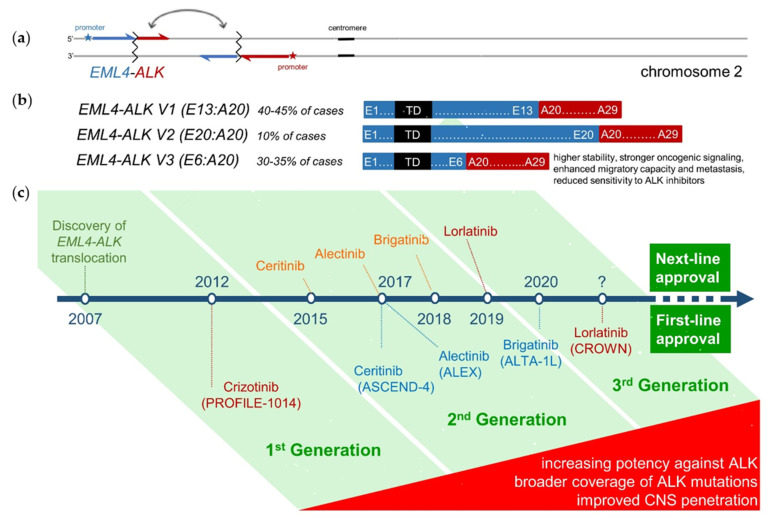
(**a**) Molecular pathogenesis of ALK^+^ NSCLC: In approximately 90% of cases, a paracentric inversion in the short arm of chromosome 2 [inv(2)(p21p23)] creates the fusion oncogene *EML4-ALK*, which facilitates overexpression of the *ALK* exons 20–29 under the influence of the *EML4* promoter, and their autophosphorylation due to the trimerization domain (TD) of EML4 [6]. In the remaining 10% of cases, a different partner gene is fused with *ALK* and triggers similar pathophysiologic effects. (**b**) The length of the *EML4* component can differ between patients, with 90% harboring *EML4-ALK* fusion variant 1, 2 or 3. Shorter variants (mainly variant 3, aka V3, E6:A20, present in 30–35% of cases) have higher stability, accumulate more and interact better with the cytoskeleton, causing stronger oncogenic signaling, reduced sensitivity to ALK inhibitors, enhanced migration and metastasis [20,21,22,23,24]. (**c**) Approval timeline of currently available ALK inhibitors, along with their respective first-line phase 3 trials. Next-generation ALK inhibitors show higher potency against the native ALK oncoprotein, broader coverage against ALK resistance mutations, and improved CNS penetration [7,8,9].

The positive results of the ALEX study marked the begin of a new era in the treatment of anaplastic lymphoma kinase-rearranged non-small-cell lung cancer (ALK^+^ NSCLC) at the end of 2017: the second-generation tyrosine kinase inhibitor (TKI) alectinib could improve both progression-free (PFS) and overall survival (OS) in a head-to-head comparison as first-line therapy vs. the first-generation compound crizotinib, thus irrevocably displacing it as the preferred upfront pharmaceutical [1,2,3]. Meanwhile, the newer second-generation inhibitors brigatinib and ensartinib in the ALTA-1L and eXalt3 studies, respectively, have also demonstrated activity comparable to that of alectinib, with brigatinib already approved as a main first-line option [4,5]. These compounds are ATP-competitive inhibitors of the ALK fusion oncoprotein, whose kinase domain (usually exons 20–29) expression and autophosphorylation are induced by the 5′-fusion-partner-gene’s (usually *EML4*) promoter and oligomerization domains, respectively (Figure 1a,b) [6]. Compared to crizotinib, second-generation TKI are more selective and have lower half-maximal inhibitory concentrations (IC50) for the native ALK kinase, cover more *ALK* resistance mutations, and show better central nervous system (CNS) penetration (Figure 1c) [7,8,9]. The third-generation compound lorlatinib, which was specifically designed to have higher potency against ALK, effectiveness against all known *ALK* resistance mutations, and improved brain pharmacokinetics, recently showed even better superiority vs. crizotinib in the phase 3 CROWN study, and is also expected to move into the first line soon [10]. A similar trend for earlier use of more potent drugs is currently noted in EGFR-mutated (EGFR^+^) NSCLC as well, where osimertinib could recently displace earlier TKI as initial treatment for tumors with exon 19 deletions or the L858R mutation [11]. However, the triumph of the “best-first” approach in ALK^+^ is much more impressive than in EGFR^+^ disease, almost “total”, based on two unique characteristics: first, clear superiority vs. sequential therapies, since the median PFS of upfront alectinib/brigatinib, namely 2.5–3 years, exceeds by at least 1 year the combined PFS of sequential treatment with first-line crizotinib followed by alectinib or brigatinib [12,13,14,15,16,17], something which upfront osimertinib cannot demonstrate vs. the sequence afatinib-osimertinib [11,18]; and second, the potentially 100% rate of subsequent targeted therapy for patients progressing under any first-line ALK inhibitor, because the approval status of next-line TKI is not tied to specific molecular results, like presence of *EGFR* T790M in patients failing EGFR inhibitors. From a biological view, this spectacular success is presumably related to the very low total number of missense mutations present in the exome of ALK^+^ tumors (tumor mutational burden, TMB), uniquely <3 mutations per Mb on average, which is lower than in all other NSCLC, indicates a higher genetic stability, and presumably facilitates the longer retainment of oncogene-addiction under targeted pharmaceuticals [19]. The principles behind management of ALK^+^ tumors today build the focus of the current work.

### 1.1. Best-First for Longer Survival

Main compelling arguments for upfront use of alectinib and brigatinib today are their greater systemic efficacy compared to crizotinib, with a PFS hazard ratio of approximately 0.5 (Table 1), as well as an even larger advantage regarding intracranial disease control, with a rate of brain progression <10% vs. >20% annually for crizotinib (Table 2) [1,5]. In contrast, if these drugs are given after crizotinib, they lose most of their systemic efficacy, with a PFS drop of 17 months for alectinib [13,14] and >12 months of brigatinib [15,16], which is longer than the entire first-line PFS of crizotinib (Table 1). Second-line brain efficacy is also reduced, with a brain ORR for alectinib and brigatinib about 65%, which is considerably lower than the 80% noted in the first line (Table 2). Furthermore, with first-line crizotinib, an additional 15–20% of patients will experience brain progression under treatment before they even get to receive second-generation compounds, based on a rate of CNS progression >20% annually with crizotinib vs. <10% with the next-generation compounds (Table 2) [25]. Even though the rate of brain involvement at initial diagnosis of ALK^+^ lung cancers is similar to that of other NSCLC, namely approximately 25% [26,27], brain control is especially important for ALK^+^ patients, because they are generally younger and have a longer life expectancy, wherefore quality of life matters more.

Meanwhile, the rationale behind upfront use of second-generation ALK inhibitors has been consolidated by a statistically significant OS benefit with alectinib vs. crizotinib in the ALEX trial [28]. This achievement becomes more remarkable, if one considers that crizotinib itself could not directly demonstrate an OS advantage over chemotherapy in the final analysis of the PROFILE 1014 study [29], and that the majority (53.5%) of crizotinib-treated ALEX patients did in fact receive newer ALK inhibitors subsequently [28]. Therefore, not only the mere use of next-generation ALK pharmaceuticals, but also the timing of their administration appears to have critical importance in order to exploit their full potential. Several other lines of evidence also suggest that using these novel drugs upfront is essential for long-term benefit: for example, in the 2014 analysis of the ASCEND-1 trial, the PFS curve of patients who received upfront ceritinib flattened at about 50%, suggesting long-lasting benefit over 2 years in a considerable fraction of patients, while the PFS curve of patients treated with ceritinib after previous crizotinib decreased continuously, suggesting that the responses lacked durability [30]. The 2016 update of the same trial confirmed this phenomenon [31], which is also evident in the PFS curves of first- vs. second-line alectinib [1,14] and brigatinib trials [5,16]: in all cases, upfront administration of next-generation ALK TKI facilitates a plateau of the PFS curve at a higher level than if the same pharmaceutical is administered after crizotinib. The durability of responses as a distinguishing feature for the upfront use of next-generation ALK inhibitors over crizotinib, can nicely also be seen in the two arms of the ALEX trial, the PFS curves of which run similar for the first 5.5 months, but show an increasing deviation in favor of alectinib afterwards [1,32]. In the more recent ALTA-1L and CROWN studies, this curve separation in favor of the newer compounds brigatinib and lorlatinib vs. crizotinib begins even earlier, after approximately 3.5 months [5,10].

### 1.2. Best-First before Chemotherapy

Along the same lines, there is some evidence that upfront administration of chemotherapy may also shorten the duration of TKI responses in ALK^+^ NSCLC. The final results of the PROFILE-1014 trial, published in 2018, showed a difference of approximately 20% in the 5-year OS of patients who had initially received crizotinib vs. these who had initially received chemotherapy, despite the fact that 87% of the latter subsequently received at least one ALK inhibitor, as well [29]. In keeping with this, in the first report of the PROFILE-1014 trial in 2014 [33], there was a similar difference of about 20% in the PFS after 2 years (i.e., in the tail of the PFS curve) between patients who received crizotinib vs. chemotherapy as the first systemic treatment; but no such difference was evident when crizotinib and chemotherapy were both given after first-line chemotherapy in another study [33], despite very similar PFS HRs in favor of crizotinib in the two studies (0.45 in PROFILE-1014 vs. 0.49 in the other study). Overall, these observations suggest an impairment of TKI benefit after exposure to chemotherapy. This could be due to the genotoxic effect of the chemotherapy [34], since accumulation of genetic abnormalities can facilitate TKI escape in human patients and preclinical models [35,36]. It is tempting to speculate that this putative disadvantage from early exposure to chemotherapy might be even more important for ALK^+^ lung cancers compared to other NSCLC, because of the particularly low baseline TMB of ALK^+^ disease [19]. Based on these considerations, the results of molecular testing, including ALK status, should generally be awaited instead of “blindly” starting chemotherapy for metastatic NSCLC, and available TKI treatment options should generally be exhausted before resort to cytostatics. The latter is an important principle of therapeutic sequencing, as will be detailed in the following sections.

### 1.3. When Should Brain Radiotherapy Be Offered?

Whether brain radiotherapy should be offered upfront together with targeted therapies for newly diagnosed oncogene-driven NSCLC featuring CNS involvement has been a matter of controversy [37]. A retrospective analysis of EGFR^+^ NSCLC patients with brain metastases published in 2017 by Magnuson et al. could demonstrate a survival advantage for the addition of cerebral irradiation to first-/second-generation EGFR inhibitors, which has been a strong argument for the combined approach [38]: in this study, the rate of brain progression was slightly lower for patients treated with TKI and stereotactic (SRT) or whole-brain radiotherapy (WBRT) compared to TKI alone, which also translated to a significant OS benefit in the long run. However, in absolute terms the rate of brain progression in all three patient groups (i.e., TKI-only, TKI and SRT, TKI and WBRT) was >20% per year, comparable to that observed in the control (erlotinib or gefitinib) arm of the FLAURA trial, and much higher than the <10% annually observed with osimertinib in the experimental arm of the same study [39]. Hence, upfront osimertinib alone protects against brain progression much better compared to older EGFR TKI plus radiotherapy, which should therefore be probably reserved as salvage treatment at the time of osimertinib failure. As already discussed, in ALK^+^ disease, first-line alectinib or brigatinib are also associated with a similar, very low rate of brain progression < 10% per year (Table 2), which argues for a similar strategy (Table 2). Furthermore, even though the FLAURA, ALEX and ALTA-1L studies had included only patients with asymptomatic brain lesions (Table 2), real-world data suggest that the same principles probably hold true for large (>1 cm) or symptomatic brain metastases, as well: a retrospective analysis of such tumors treated with alectinib in the routine setting showed an efficacy comparable to that observed with TKI for smaller, asymptomatic lesions in the aforementioned clinical studies [40]. Overall, available data argue for a “radiation-free” first line with the newer ALK pharmaceuticals, however one important exception warrants special attention: patients diagnosed with oligometastatic disease to the brain, for whom treatment has a curative intention, should always receive additional, consolidative radiotherapy, similar to the standard practice in case of extracranial oligometastases [41]. For other patients, surveillance using longitudinal brain magnetic resonance imaging (MRI) emerges as the preferable option, with SRT to be offered as salvage treatment at the time of brain progression, because the brain response rate with any next-line ALK inhibitor or chemotherapy does not exceed 60–70% (Table 2), i.e., TKI brain efficacy for ALK inhibitors beyond the first line is roughly similar to that of first-line erlotinib/gefitinib, for which administration of additional radiotherapy improved OS in the Magnuson et al. study [38]. Of note, SRT is meanwhile feasible for up to 10 or even more brain lesions with excellent outcome [42,43], therefore use of the neurotoxic WBRT [44] should be strongly discouraged for ALK^+^ NSCLC, as long as potentially effective systemic treatments are still available.

### 1.4. Safety and Tolerability

**Figure 2:**
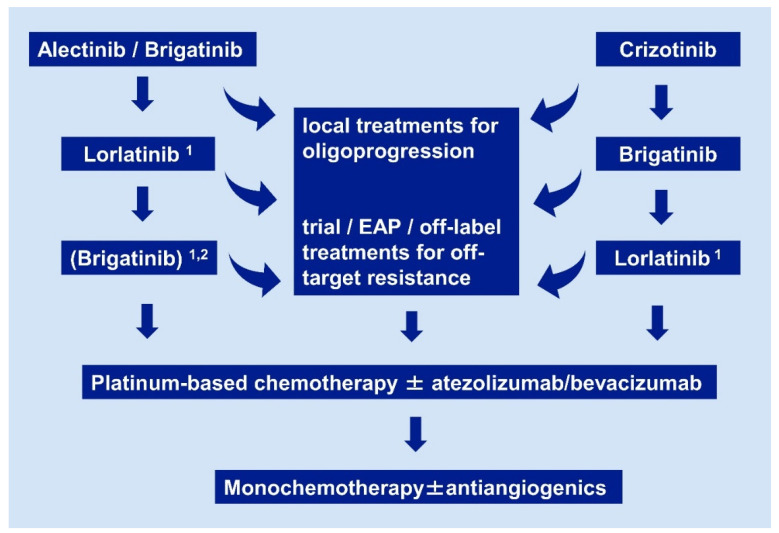
Current algorithm for sequential use of systemic pharmaceuticals in ALK^+^ NSCLC. Details and the rationale are given in Section 2.1, Section 2.2, Section 2.3, Section 2.4 and Section 2.5. Footnotes: ^1^ if no ALK mutation is detectable by molecular analysis of tissue or liquid rebiopsies, close monitoring with short-term restaging after ≤ 6 weeks is warranted, in order to ensure timely switch to chemotherapy if there is no response; ^2^ a trial of brigatinib after lorlatinib for patients who did not receive brigatinib as first-line treatment in order to prolong chemotherapy-free time, based on the results of the international EAP, with a median duration of treatment 7.5 months for the lorlatinib-pretreated cohort [70]. EAP: extended access program.

Safety and tolerability are generally very good for most ALK inhibitors and of minor importance for the selection of initial or subsequent treatment. For alectinib, treatment-related adverse events occurring in ≥5% of patients are only elevated liver enzymes and anemia [1], while for brigatinib CPK increase and hypertension [5]. Of note, even the early-onset pulmonary events, which were observed in 8% of patients receiving brigatinib in later treatment lines and worrisome in the ALTA trial, are rare (3%) in the first-line setting [45]. Overall, the frequencies of adverse events leading to dose reduction were comparable for alectinib, brigatinib or lorlatinib vs. crizotinib in the ALEX, ALTA-1L and CROWN studies [1,5,10], while the percentage of patients with treatment discontinuation was approximately 10% and similar across the control and experimental arms of all three trials. One exception to this rule of good tolerability for ALK inhibitors is the second-generation compound ceritinib, which shows considerable gastrointestinal toxicity, i.e., not only elevation of liver enzymes in approximately 20% of cases, but also grade 3 nausea/vomiting and diarrhea in 5–8% of patients, worse than chemotherapy in the randomized phase 3 ASCEND-5 study [46,47]. Changing the standard ceritinib dose to 450 mg with food instead of 750 mg fasted, based on the results of ASCEND-8 [48], and proactive management [49], have meanwhile mitigated these problems, but ceritinib is also the weakest second-generation ALK pharmaceutical and does not play an important role in therapeutic algorithms any more (Table 1 and Figure 1c and Figure 2). In contrast, the distinct side-effect profile of the third-generation TKI lorlatinib warrants special attention, because this compound is the standard treatment after failure of any second-generation inhibitor, and also expected to move in the first line soon [10]. Most frequent adverse events under lorlatinib are hypercholesterolemia and hypertriglyceridemia, occurring in 70% and 64% of patients, respectively, which are usually asymptomatic and readily managed with lipid-lowering agents and dose modifications. Other notable side effects in the CROWN study were edema (55%), weight gain (38%), as well as cognitive (21%) and mood disturbances (16%), which occurred typically in the first 2 months of treatment, were mostly low grade, and could successfully be managed with treatment interruptions and dose reductions. Weight gain as well as neuropsychiatric changes may be due to off-target inhibition of tropomyosin receptor kinase B in the CNS [50,51]. Considering the excellent efficacy of lorlatinib in the CROWN study (Table 1), its particular toxicity is expected to play a minor only role in therapeutic decision-making. Notwithstanding, hyperlipidemia and the occasional neuropsychiatric symptoms are certainly more worrisome in the upcoming first-line lorlatinib setting, with an expected treatment duration and life expectancy of several years, than with current use of lorlatinib for late-stage patients with a median PFS below one year (Table 1).

## 2. State-of-the-Art after the First Line

### 2.1. Oligoprogressive Patients

Local treatments, mainly radiotherapy and surgery, are generally much more drastic against lung cancer cells than currently available systemic treatments. At the same time, anatomically restricted treatment failure, i.e., “oligoprogression” (OPD), is relatively frequent in oncogene-driven NSCLC, affecting at least 30–50% of patients in several series, which is clearly more than the approximately 15% of immunotherapy-treated patients, or the anecdotal only occurrence under chemotherapy [52,53]. The therapeutic importance of OPD stems from the opportunity to eradicate resistant tumor clones with ablative measures, for example surgery, stereotactic radiotherapy, cryotherapy or radiofrequency ablation, in a driver-agnostic manner. Indeed, oncogene-driven NSCLC has been a model disease for the study and therapeutic exploitation of OPD, proper management of which has resulted in a median time-to-next-treatment gain of 5–10 months and OS prolongation in several series [53,54,55,56,57,58]. Of key importance for this purpose is state-of-the-art imaging, ideally with positron emission tomography–computed tomography (PET-CT) and brain MRI, which have a higher sensitivity than CT for the identification of suitable cases [59,60]. A switch of systemic pharmaceuticals for ALK^+^ NSCLC becomes necessary only after local ablative options have been exhausted.

### 2.2. Systemic Treatment after Alectinib or Brigatinib

Currently, only the third-generation TKI lorlatinib is approved for the treatment of ALK^+^ NSCLC patients progressing under second-generation ALK inhibitors. This is based on the results of a global phase 2 study showing an objective response rate (ORR) of 32–39%, and a median PFS of 5.5–6.9 months in the respective subcohorts (EXP3B/4/5, Table 1) [61]. For the subset of patients with detectable *ALK* resistance mutations in tissue samples before the start of lorlatinib, efficacy was twice as high compared to negative patients, with an ORR of 69% and a median PFS of approximately 11 months [62]. The most frequently encountered *ALK* mutation in this setting is p.G1202R (G1202R), which occurs in at least one-third of cases [62], and can be inhibited by clinically achievable concentrations of lorlatinib in most patients [63], but not by alectinib or earlier ALK inhibitors (Figure 1c) [35]. For brigatinib, its potential clinical utility after alectinib is currently under investigation, with an ORR of 42% (*n* = 19) and a median PFS of 6.4 months in a small investigator-initiated (IIT) phase 2 trial (NCT02706626) [64,65], while results of the much larger (*n* = 104) Takeda-sponsored phase 2 Brigatinib-2002 (ALTA-2) study (NCT03535740) are still pending. Interestingly, brigatinib has also demonstrated considerable preclinical activity against p.G1202R [66], which is probably sufficient for effective inhibition in vivo, when the higher dose of 180–240 mg/day is used, as directly tested by the ongoing ALTA-2 study (NCT03535740).

### 2.3. Systemic Treatment after Crizotinib

After first-line treatment with crizotinib, all ALK inhibitors have shown clinically relevant activity, but the best results have been achieved with brigatinib, which resulted in a median PFS exceeding 1 year in two phase 2 studies (Table 1). In contrast, the median PFS was approximately 6 months with ceritinib, 8–9 months with alectinib, and 11 months with lorlatinib in the post-crizotinib setting (Table 1). Beyond its lower efficacy, ceritinib has also serious side-effects, as described in Section 1.4, which additionally limit its use. On the other hand, alectinib has no tolerability issues, but does not cover several *ALK* resistance mutations (e.g., p.I1171N, p.I1171S, p.L1196M [35]) as well as newer pharmaceuticals. Regarding lorlatinib, strictly speaking it not approved by the FDA or the EMA after treatment with crizotinib only [67,68], but requires failure of at least one additional, second-generation inhibitor. Thus, there are several reasons for preferential use of brigatinib in the second-line, post-crizotinib setting. Of note, *ALK* resistance mutations are detectable in less than 1/3 of tumors progressing under crizotinib, and their presence or absence does not correlate with benefit from subsequently used ALK inhibitors [69].

### 2.4. Treatment with ALK Inhibitors after Lorlatinib

Lorlatinib-resistant ALK^+^ NSCLC represents a major challenge and unmet clinical need currently. Older TKI are generally not helpful for these patients, because lorlatinib has the greatest potency against the ALK kinase, and the broadest activity against *ALK* resistance mutations among all currently available ALK inhibitors (Figure 1c) [10,35]. One potential exception is brigatinib, which has a slightly better activity than lorlatinib for the p.G1269A mutation [66] and produced clinically relevant benefit with a median time-to-next-treatment of 7.5 months in the lorlatinib-pretreated subcohort of the international expanded access program (including mostly European patients, Table 1 and Figure 2) [70], as well as in several case reports (e.g., [71] and patient 28 in [72]). Of note, due to the good activity of lorlatinib against all individual *ALK* mutations analyzed so far, on-target lorlatinib resistance usually results from compound (i.e., multiple coexistent) *ALK* mutations, which also confer resistance to all other currently available ALK TKI [73,74]. One promising prospect for these cases is the novel, fourth-generation macrocyclic ALK TKI TPX-0131, which has a more compact structure, less susceptibility to steric hinderance, higher potency against the gatekeeper p.L1198F, solvent-front p.G1202R and compound *ALK* mutations than currently available compounds, and is expected to enter clinical testing soon [75]. 

**Table 1:** Systemic efficacy of ALK inhibitors upfront and as next-line treatment for ALK^+^ NSCLC.

**First Line:**	**Crizotinib**	**Ceritinib**	**Alectinib**	**Alectinib**	**Brigatinib**	**Ensartinib**	**Lorlatinib**	**Lorlatinib**
study [ref.]	PROFILE-1014 [12,29]	ASCEND-4 [46]	J-ALEX [76,77]	ALEX [1,28]	ALTA-1L [5,78]	eXalt3 [4]	CROWN [10]	global phase II [61] (EXP1)
comparator	chemo	chemo	crizotinib	crizotinib	crizotinib	crizotinib	crizotinib	single arm
patients (*n*)	172	189	103	152	137	143	149	30
ORR (%)	74	73	76	83	76	75	76	90
mPFS (mo)	10.9	16.6	34.1	25.7 */34.8 **	24.0 */29.4 **	25.8*	NR	NR
HR	0.45	0.50	0.34	0.50	0.49	0.52	0.28	N/A
**Post Crizotinib:**	**Ceritinib**	**Ceritinib**	**Alectinib**	**Alectinib**	**Brigatinib**	**Brigatinib**	**Ensartinib**	**Lorlatinib**
study [ref.]	ASCEND-1 [31]	ASCEND-2 [79]	global phase II [13]	phase II [14]	phase I/II [15]	ALTA 90/180 mg [16,17]	phase I/II [80]	global phase II [61,62] (EXP2/3A)
patients (*n*)	163	140	138	87	70	110	29	59
ORR (%)	56	38	50	48	71	55	69	70
mPFS (mo)	6.9	5.7	8.9	8.1	13.4	12.9/16.7	9	11.1
**Post Alectinib (or other 2G TKI):**		**Ceritinib**	**Ceritinib**	**Brigatinib**	**Brigatinib**	**Brigatinib**	**Brigatinib**	**Lorlatinib**
study [ref.]		phase II [81] (Japan)	retrospective [82] (Japan)	ALTA-2 [83]	Phase II (IIT, USA) [64]	retrospective [65]	EAP (EU) [70]	global phase II [61,62] (EXP3B/4/5)
patients (*n*)		20	9	103	19	18	111	139
ORR (%, (*n*))		25 (4/20)	44 (4/9)	pending	47 (9/19)	17 (3/18)	n/a	41 (52/127)
mPFS (mo)		3.7	4.4		6.4	4.4	n/a	6.9
mTNT (mo)							8.7	
**Post Lorlatinib:**					**Brigatinib**			
study [ref.]					EAP (EU) [70]			
patients (*n*)					37			
ORR (%)					n/a			
mPFS (mo)					n/a			
mTNT (mo)					7.5			

2G TKI: second-generation tyrosine kinase inhibitor; ORR: objective response rate; mPFS: median PFS; mo: months; HR: hazard ratio; * IRC (independent review committee) assessed; ** INV (investigator)-assessed; NR: not-reached; n/a: not available; mTNT: median time to next treatment or discontinuation of treatment; IIT: investigator-initiated trial; [ref.]: reference of this manuscript; EU: the EAP included mostly European patients.

**Table 2:** Brain efficacy of ALK inhibitors and other options upfront and as next-line treatment.

**ALK^+^, First Line:**	**Crizotinib**	**Ceritinib**	**Alectinib**	**Brigatinib**	**Ensartinib**	**Lorlatinib**
study [ref.]	ALTA1L-ALEX (asympt. BM) [1,5]	ASCEND-4 (stable BM) [46]	ALEX (asympt. BM) [1]	ALTA-1L (asympt. BM) [5]	eXalt3 (asympt. BM) [4]	CROWN (asympt. BM) [10]
patients (*n*) for iORR:	21–22	22	21	18	13	14
iORR (%)	29–50	73	81	78	75	82
patients (*n*) total:	138–151	n/a	152	137	143	149
brain progression at 1 year:	19–41	n/a	9.4	8.8	4	2.8
**ALK^+^, Post Crizotinib:**		**Ceritinib**	**Alectinib**	**Brigatinib**	**Ensartinib**	**Lorlatinib**
study [ref.]		ASCEND-2 [79]	phase I/II pooled [84]	ALTA (180mg) [85]	phase I/II [80]	phase II [61]
patients (*n*)		20	50	18	6	37
iORR (%)		45	64	67	83	68
iORR (%) 1L		73	81	78	75	75
ΔiORR 2L-1L		−29%	−17%	−11%	8%	−7%
(% of 1L)		(−39%)	(−21%)	(−14%)	(+11%)	(−9%)
**EGFR^+^, First Line and beyond:**		**Gefitinib/Erlotinib**		**Osimertinib**		**Radiotherapy /+ΤΚΙ**
study [ref.]		FLAURA [39] (stable BM)		FLAURA [39] (stable BM)		various studies [86,87,88]
patients (*n*) for iORR:		19		22		
iORR (%)		68		91		50–60 / 85
patients (N) total:		277		279		
brain progression at 1 year:		24		8		

BM: brain metastases; asympt.: asymptomatic; iORR: intracranial objective response rate; ΔiORR: difference in iORR; 1L: first-line; n/a: not available; ΤΚΙ: tyrosine kinase inhibitors.

### 2.5. Do We Need Rebiopsies and Next-Generation Sequencing (NGS) Testing?

Even though available data about ALK TKI in the next-line setting can be summarized in an unambiguous empirical algorithm (Figure 2), and the approval status of these drugs does not depend on the detection of any specific molecular alteration in rebiopsies, profiling of ALK^+^ NSCLC at progression is still very desirable, mostly for one main reason: the identification of potentially actionable off-target resistance mechanisms. A few examples include *MET* amplifications, *HER2* amplifications, and *KRAS* mutations [89,90,91,92]. These can emerge under treatment with any ALK inhibitor, including lorlatinib [93], and their detection is essential, because they are generally resistant to ALK TKI, but potentially sensitive to other available drugs. As one notable exception, *MET* amplifications are sensitive to the first-generation ALK inhibitor crizotinib and represent in fact the only relevant indication for this compound in ALK^+^ NSCLC today. In contrast to the crucial importance of off-target resistance, typing of *ALK* resistance mutations has meanwhile limited clinical utility. Originally, one main intention behind the mapping of IC50 for each individual *ALK* mutation and available ALK inhibitor, was to facilitate “TKI-sparing”, i.e., use of the ALK inhibitor with the narrowest possible spectrum according to the molecular results in each patient, similar to antibiotic stewardship for infectious diseases: for example, if *ALK* p.L1198F or *ALK* p.L1196M was detected in a patient failing the second-generation TKI alectinib, this patient could subsequently receive crizotinib or ceritinib respectively, which show very low IC50 for these mutations, so that more potent substances remain available as future options. However, viewed from today, this approach makes little sense, because the “best-first” strategy has already proven superior to TKI-sparing regardless of whether any resistance mutations are present, namely even in the treatment-naive upfront setting. Moreover, the detection of a specific *ALK* mutation in tissue or liquid biopsies cannot exclude presence of additional tumor clones with other *ALK* mutations in the same patient, which might be resistant to earlier ALK inhibitors. Therefore, lorlatinib is always preferable after failure of second-generation inhibitors, regardless of which *ALK* mutation is present. In addition, a trial of lorlatinib treatment after second-generation AKI TKI is warranted even in patients without detectable *ALK* mutations, because up to 20% of them can show responses lasting > 1 year, as observed in the phase 2 trial [62]. However, caution is warranted in these cases, with close patient monitoring, so that the switch to chemotherapy is not delayed in case of further tumor progression with imminent clinical deterioration (Figure 2). Similar applies to patients with detection of multiple *ALK* resistance mutations: these might be originating from different tumor cell clones and do not necessarily correspond to compound *ALK* mutations, therefore they should not be used to exclude patients from a trial of lorlatinib. Even more challenging are cases with off-target resistance when suitable targeted compounds are not available (e.g., *KRAS* mutation, *HER2* amplification): despite presence of the additional alteration, in some cases the disease remains ALK-dependent and the switch from second-generation ALK TKI to the more potent lorlatinib can facilitate tumor responses lasting several months (unpublished personal observation of the authors). Finally, a therapeutic dilemma also ensues when *ALK* p.G1202R is detected directly after crizotinib, which can occasionally occur in 5–10% of cases [69]. Considering that brigatinib has also clinically relevant activity against *ALK* p.G1202R, as explained in Section 2.1, and that lorlatinib can also be given after brigatinib, a trial of brigatinib treatment is a reasonable choice for these patients before switch to lorlatinib, in accordance with the proposed empirical algorithm (Figure 2).

### 2.6. The Emerging Concept of Molecular Risk and Value of Disease Monitoring in ALK^+^ NSCLC

Beyond identification of resistance mechanisms which are important for the choice of next-line pharmaceuticals, NGS also facilitates deeper biological understanding of the ALK^+^ disease, which is increasingly influencing patient management. Accumulating data from several retrospective analyses [23,24,94,95,96,97] and the randomized phase 3 study ALTA-1L [98] show that the “short” *EML4-ALK* fusion variant 3 (E6:A20, Figure 1b) is associated with earlier treatment failure and shorter survival regardless of the type of treatment. V3-positive cases comprise approximately one-third of all ALK^+^ NSCLC and present with more aggressive disease already at baseline, as evident by a higher number of metastatic sites at initial diagnosis [22,23,24,99]. Biochemical basis for this adverse clinical phenotype is the higher stability of shorter *EML4-ALK* variants, which leads to accumulation and stronger oncogenic signaling, as well as their better interaction with the cytoskeleton, which increases the migratory capacity of V3-positive cancer cells [22,100]. Of note, instead of the DNA-NGS used in negative studies [32], investigators who detected differences in patient outcome according to the *EML4-ALK* fusion variant have generally relied on RNA-based methods, mainly RNA-NGS [23,95,101] and RT-PCR [23,24], which are more suitable for the detection of oncogenic fusions in lung adenocarcinoma [102]; or have used particularly sensitive ctDNA assays, like ctDx-Lung in case of the ALTA-1L analysis (personal communication with Takeda), which outperformed the Guardant 360 assay (itself equivalent to standard tissue genomic testing [103]) in a comparative study [104]. In fact, the prominent impact of *EML4-ALK* fusion variant on the outcome of both brigatinib and crizotinib-treated patients in the ALTA-1L study [98], suggests that the negative results of a similar analysis for both crizotinib and alectinib-treated patients in the ALEX study [32] are probably inaccurate. One possibility is that the less sensitive DNA-NGS used in the ALEX analysis might have missed some variant 1 (V1, E13:A20, Figure 1b)-positive cases with lower allelic fractions, which generally have more favorable courses, thus blunting the difference between V1 and V3. Furthermore, presence of *TP53* co-mutations has been highlighted as an additional independent molecular risk factor for adverse outcome in multivariable analyses of the ALTA-1L and an earlier real-world study, so that “double-positive”, i.e., V3^+^*TP53*^mut^ patients represent a “very-high-risk” subset with a median overall survival of approximately 2 years currently [98,105]. Of note, beyond the approximately 25% of ALK^+^ patients with *TP53* mutations present already at baseline [105,106], another 20–25% will acquire *TP53* mutations at the time of disease progression, which is also associated with poor survival similar to that of primarily *TP53* mutated cases [107]. These data demonstrate the ability of NGS results to predict the outcome of individual ALK^+^ NSCLC patients with increasing accuracy. This patient-specific “molecular risk” in ALK^+^ NSCLC appears to impair efficacy of all treatments analyzed so far, and cannot therefore be used for selection of specific pharmaceuticals yet [19]. However, improved prognostication can affect patient management in several other ways, for example it can assist selection of unfavorable cases for more aggressive surveillance and local treatment strategies, or for preferential participation in early phase clinical trials. Luckily, inasmuch as high-risk V3-positive and/or *TP53*-mutated cases need closer monitoring, they also have a higher number and higher abundance (variant allele frequencies) of molecular alterations in the blood circulating tumor DNA (ctDNA), which can be used for remission tracking and earlier identification of treatment failure [72,91]. Besides single-nucleotide variants (SNV), copy number alterations, as captured globally by low-pass (0.5–1x) whole-genome sequencing and quantified with the trimmed median absolute deviation from copy number neutrality (tMAD) score across the genome, are also increased in high-risk cases, accumulate further during the course of the disease, and correlate independently with the risk of death [72]. It is remarkable that longitudinal changes of the tMAD reflect the tumor remission status similar to longitudinal SNV changes, and can therefore be used for non-invasive disease monitoring in patients without detectable point mutations in ctDNA [72,91].

### 2.7. Treatment after TKI

Despite the impressive success of ALK inhibitors, with a median OS exceeding five years for patients receiving upfront alectinib [28], metastatic ALK^+^ NSCLC remains incurable, and TKI resistance eventually develops in all cases. For those still capable of tolerating intensive therapies, the quadruple combination of carboplatin-paclitaxel-bevacizumab-atezolizumab (IMpower150 regime) appears to be highly effective with objective responses in >50% of EGFR^+^/ALK^+^ tumors, and represents the only meaningful immunotherapy (IO) for these patients [108]. As several earlier series and preclinical studies have shown, *ALK*- and *EGFR*-driven tumors have an immunosuppressive tumor microenvironment with resistance to programmed cell death protein 1 (PD-1) and programmed cell death 1 ligand 1 (PD-L1) inhibitors [109,110]. However, the synergy of the VEGF-inhibitor bevacizumab with the PD-L1 inhibitor atezolizumab appears to overcome the IO-resistance of oncogene-driven NSCLC, which is in part mediated by VEGF induced by oncogenic signaling [111]. On the other hand, several clinical trials of PD-1/PD-L1 inhibitors in combination with ALK TKI have failed, showing only increased toxicity, but no efficacy gain [112]. Further options for TKI-refractory ALK^+^ NSCLC patients currently include platinum-based doublets, especially with pemetrexed, and monochemotherapy alone or in combination of antiangiogenics (docetaxel in combination with nintedanib or ramucirumab) [113,114,115]. However, efficacy is modest and median survival does not exceed 12–15 months according to retrospective studies [116,117]. An exciting prospect is the ability to target ALK-derived neopeptides using autologous or allogeneic T-cell receptor (TCR)-transgenic T-cells, based on the fact that anti-ALK T-cell responses are readily detectable in normal donors and many ALK^+^ NSCLC patients [118,119]. The TCR-therapy field has been revolutionized by several technological breakthroughs during the last few years [120], so that these concepts are expected to enter early clinical testing soon. The development of effective immunotherapies, and particularly cell therapies, is the single most important pharmaceutical challenge for ALK^+^ NSCLC today, since no other systemic treatment, including TKI, has so far been able to eradicate these tumors.

## 3. Conclusions

ALK^+^ NSCLC is a model disease for the management of OPD, use of targeted pharmaceuticals, and clinical utility of advanced molecular profiling in thoracic oncology. Standard first-line treatments are currently the second-generation ALK inhibitors alectinib and brigatinib. Lorlatinib and brigatinib are the preferred next-line therapies after progression under second-generation TKI and crizotinib, respectively, except for cases with off-target resistance detected in tissue or liquid rebiopsies, which require special therapeutic maneuvers. Presence of the *EML4-ALK* fusion variant 3 and/or *TP53* mutations identify high-risk cases with earlier treatment failure and shorter survival, regardless of the applied therapy. The potential clinical utility of serial ctDNA assays for improved guidance of treatment in these cases is currently a subject of intense investigation. Major pharmaceutical challenges for the field are the development of more potent, fourth-generation TKI and effective immunotherapies, which will be essential in order to cure ALK^+^ NSCLC in the future.
